# Utilizing the Postvascular Phase of Contrast-Enhanced Ultrasound to Predict Breast Cancer Lymph Node Metastasis

**DOI:** 10.3390/medicina60111780

**Published:** 2024-10-31

**Authors:** Jiuyi Ma, Ying Fu, Xiangmei Chen, Yuxuan Lin, Lan Zeng, Fang Mei, Ligang Cui

**Affiliations:** 1 Department of Ultrasound, Peking University Third Hospital, 49 North Garden Road, Haidian District, Beijing 100191, China; 2 Department of Ultrasound, Peking University Shenzhen Hospital, Shenzhen 518036, China; 3 Department of Ultrasound, Beijing Friendship Hospital, Capital Medical University, Beijing 100050, China; 4 Department of Pathology, Peking University Third Hospital, 49 North Garden Road, Haidian District, Beijing 100191, China

**Keywords:** breast neoplasms, contrast media, lymphatic metastasis, Sonazoid, ultrasonography

## Abstract

*Background and Objectives*: To evaluate the value of the postvascular phase of contrast-enhanced ultrasound (CEUS) in differentiating between benign and metastatic lymph nodes (LNs) in patients with breast cancer (BC). *Materials and Methods*: This study retrospectively analyzed 96 suspicious LNs in the lymphatic drainage area of the breast from 90 patients with BC. All LNs were assessed by conventional ultrasound (US) and CEUS following intravenous Sonazoid injection. All LNs underwent puncture biopsy, and pathological results were obtained. The correlations between US and CEUS indicators of LNs and LN metastasis (LNM) were analyzed. *Results*: Of the 96 LNs, 66 were metastatic. Overall, 80.00% (24/30) of the benign LNs exhibited relative hyper-enhancement in the postvascular phase, whereas 96.97% (64/66) of the metastatic LNs exhibited relative hypo-enhancement (*p* < 0.001). This CEUS finding was highly predictive of metastasis, with a sensitivity of 96.97%, specificity of 80.00%, positive predictive value of 91.43%, negative predictive value of 92.31%, and accuracy of 91.67%. The mean postvascular phase intensity (MPI) was significantly lower for malignant (median MPI, 12 dB) than for benign (median MPI, 75 dB) LNs. The postvascular phase was more sensitive, specific, and accurate than conventional US or the vascular phase of CEUS for the diagnosis of LNM, with an area under the curve of 0.95 (95% confidence interval: 0.89–0.99). *Conclusions*: Qualitative and quantitative indicators of the postvascular phase of CEUS provide a reliable diagnostic approach to differentiate benign and metastatic LNs in patients with BC.

## 1. Introduction

Histological type is a critical determinant of prognosis in breast cancer (BC) prognosis, but lymph node (LN) status also helps in management decisions regarding adjuvant therapy. The 5-year survival rate for patients with lymph node metastasis (LNM) is markedly lower than that for those with lesions confined to the breast [1,2]. Sentinel lymph node biopsy (SLNB) has recently replaced full axillary lymph node dissection (ALND) without compromising survival in patients with two or fewer metastatic sentinel LNs [3]. Consequently, SLNB is generally used to assess the pathological status of LNs; however, standard SLNB requires precise localization of sentinel LNs using methylene blue or radionuclides prior to excision, a procedure that is both complex and time-consuming [4,5]. Thus, an efficient assessment of axillary nodal status is crucial for BC staging.

Conventional ultrasound (US) is increasingly utilized for this purpose, with a sensitivity of 26–76% based on morphological criteria, but it is inadequate for accurately determining LNM [2,6]. For suspected LNs, US-guided biopsy remains a convenient, rapid, and widely used method. A meta-analysis reported that US-guided core-needle biopsy (CNB) and fine-needle aspiration (FNA) of axillary nodes had a sensitivity of 88% (95% confidence interval [95% CI]: 84–91%) and 74% (95% CI: 70–78%), respectively, with both demonstrating a specificity of 100%, for diagnosing LNM in BC patients [7]. Compared to FNA, CNB’s sensitivity significantly improved but did not achieve a sensitivity of 90–95% of the SLNB examination, as recommended by the guidelines [8,9].

Nevertheless, puncture biopsy remains an invasive procedure. Consequently, various US techniques have been developed to predict LN malignancy, including diagnostic models that integrate different gray-scale indicators, elastography, and contrast-enhanced US (CEUS). Several studies have reported problems with inter-observer agreement in elastography for axillary LNs [10,11,12]. Some studies have indicated that CEUS may be an effective alternative; however, the diagnostic value of certain indicators in the vascular phase of CEUS, such as enhancement homogeneity, time to peak, and perfusion pattern, remains controversial [13,14,15,16].

Therefore, noninvasive detection tools with high sensitivity for predicting LNM are warranted. Recently, the use of the postvascular phase for diagnosing focal lesions in the liver has increased [17,18,19]. However, studies on the postvascular phase of LNs are scarce. Therefore, this study aimed to examine the diagnostic performance of the postvascular phase in distinguishing benign and metastatic LNs in BC patients.

## 2. Materials and Methods

### 2.1. Study Participants

Between March 2020 and September 2022, the medical records of 90 consecutive patients with BC confirmed on pathological examination were reviewed. All patients were women. The inclusion criteria were a definite history or clinical diagnosis of BC, US findings suggestive of suspicious LNs in the lymphatic drainage area of the breast, and a diagnosis of LNs based on histopathological findings and follow-up. Exclusion criteria included patients with lymphoma or metastasis not originating from BC, those with severe organ dysfunction, those allergic to eggs, and pregnant or lactating patients.

### 2.2. Sonographic Examination and Biopsy

All sonographic examinations were conducted by an experienced radiologist (LG.C) using a high-resolution ultrasound system (SAMSUNG RS80A and RS85 Ultrasound Systems) with a linear probe (LA4–18B) in conventional mode. After determining the location of the suspicious nodules, gray-scale US features were recorded. The system was switched to CEUS mode, with a gain of 45–55 (dB). Before imaging, a bolus injection of 0.4 mL of Sonazoid (GE Healthcare, Oslo, Norway) was administered, followed by a flush of 5 mL saline solution through an intravenous cannula placed in the antecubital vein. After injection, the enhanced imaging video was stored until 1 min for offline baseline analysis and then until the 7th min after injection to start observation again and save the image. A comparison of the two phases of CEUS is presented in Table 1. The decision algorithm for identifying the regions of interest (ROIs) was as follows: First, the ROI was utilized in the postvascular phase and was placed inside the suspicious LN. If the LN exhibited uniform enhancement, the selection of the ROI became practically unrestricted. If the enhancement was not uniform, the ROI was placed in the region with the lowest enhancement, except for Pattern IV, in which case the ROI should be situated in the area manifesting relative hyper-enhancement. The ROI was not placed in areas of persistent nonenhancement on CEUS. Time-intensity curve analysis was performed after obtaining image information using ROIs lasting from 1 to 3 s. Of note, CNB was performed in all LNs by the same radiologist (LG.C), with the puncture site selected using the same decision algorithm for ROIs. If the patient’s LN puncture result was benign and there was a need for surgery, the skin surface would be marked, and the depth, morphology and size of the LN would be recorded after CEUS. All patients were subjected to continuous monitoring for 30 min following the injection or completion of the puncture.

If the LN puncture results were benign but surgery on the axillary and breast primaries was planned within two weeks, the same radiologist (LG.C) would perform the following steps: prior to SLNB, an intradermal areola injection of a mixture of Sonazoid and methylene blue was administered to identify and localize the sentinel LN, observing the LN’s morphology, size, depth, and body marking to determine if it was the same as that previously observed via the intravenous method. If not, the case was excluded. If so, after the LNs were excised, they were immersed in saline and reassessed using the CEUS mode, including observation of their size, morphology, and enhancement pattern to ensure they matched the previous US detections.

Another proficient sonologist (Y.F.) independently assessed the results through a comprehensive review of the image data. If there was no consensus, the final determination was made through consultation between readers. The sonographers were kept blinded to the pathological outcomes.

### 2.3. Image Analysis

For conventional US, the following imaging criteria were used to define suspicious LNs as previously reported [20,21,22]: a long-to-short diameter ratio (L/S) < 2; absence of hilum; a maximum cortical thickness ≥ 3 mm; and a vascular pattern of peripheral or mixed. In the vascular phase of CEUS, enhancement homogeneity was classified as either heterogeneous or homogeneous. A perfusion defect was defined as the absence of perfusion within the LNs. The perfusion patterns of the LNs were categorized as centrifugal, hybrid, or centripetal.

**Figure 1:**
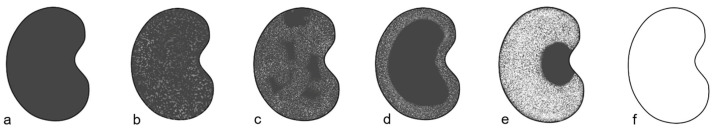
Diagram depicting enhancement patterns of lymph nodes (LNs) in the postvascular phase. (**a**) No enhancement (Pattern I); (**b**) starry hypo-enhancement (Pattern II); (**c**) heterogeneous hypo-enhancement with unenhanced area (Pattern III); (**d**) annular enhancement (Pattern III); (**e**) heterogeneous hyper-enhancement with hypo-enhancing or unenhanced areas matched with the hilum (Pattern IV); (**f**) homogeneous hyper-enhancement (Pattern V).

Consistent with the latest guidelines [23,24], the vascular phase began approximately 10–20 s after contrast injection, whereas the postvascular phase was defined at 6 min post-injection. In this study, imaging parameters in the postvascular phase were obtained at 7–9 min post-injection. In the postvascular phase, the degree of enhancement in comparison to the surrounding tissues, such as muscle tissue, was categorized as relative hypo-enhancement (Patterns I, II, and III) or relative hyper-enhancement (Patterns IV and V). Enhancement pattern of LNs can be categorized into five types (Figure 1): Pattern I: no enhancement (gradually wash-out from the vascular phase with enhancement to no enhancement); Pattern II: homogeneous or starry hypo-enhancement (diffuse point enhancement); Pattern III: heterogeneous hypo-enhancement with irregular unenhanced areas, partly manifesting annular enhancement (unenhanced area was not consistent with the hilum); Pattern IV: heterogeneous hyper-enhancement (hypo-enhancing or unenhanced area was consistent with the hilum); Pattern V: homogeneous hyper-enhancement. The mean postvascular phase intensity (MPI) [25], a quantitative parameter in the postvascular phase, was obtained from ROIs and reflected the intensity of enhancement within that region.

### 2.4. Statistical Analysis

Statistical calculations were conducted using SPSS (version 26.0) and MedCalc (version 20.0). The kappa test was used to evaluate the interreader agreement. Receiver operating characteristic (ROC) curve analysis was obtained to assess the utility of four models in differentiating benign and malignant LNs, with the DeLong test used to compare areas under the curve (AUC). A *p*-value < 0.05 was considered statistically significant.

## 3. Results

### 3.1. Patient Characteristics

**Figure 2:**
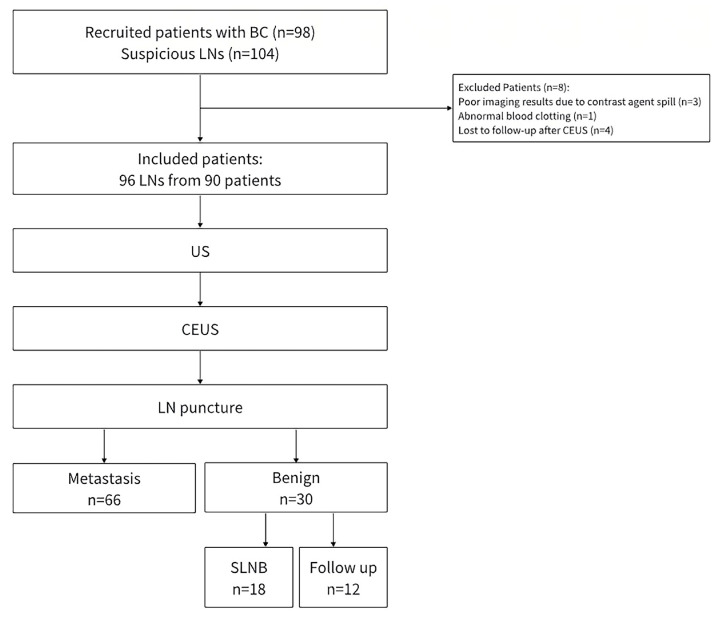
Flowchart of the study population. BC, breast cancer; CEUS, contrast-enhanced ultrasound; LNs, lymph nodes; SLNB, sentinel lymph node biopsy; and US, ultrasound.

Between March 2020 and September 2022, 90 consecutive patients (mean age, 54.44 ± 12.29 years) with 96 LNs were eligible for participation (Figure 2). Patient demographics are shown in Table 2. Malignancy was histologically confirmed through puncture, while benignity was ultimately established through surgery or follow-up. Of the 96 LN puncture results, 66 were malignant, and 30 were benign. Of the 30 benign cases, 12 underwent surgery on the primary breast lesion and axilla before enrollment, so surgical excision was not repeated this time, and they were followed up directly. No evident changes in the morphology or size of the monitored LNs were observed, with a follow-up range of 13 to 24 months (median, 17 months). The remaining 18, who had not previously had surgery for breast primaries, underwent breast surgery and SLNB at this time, all of which were sentinel LNs, and all biopsies turned out to be benign.

### 3.2. Conventional US and CEUS Examinations

The association between sonographic parameters and LN status is detailed in Table 3. Among the indicators of conventional US and the vascular phase of CEUS, an L/S < 2, cortical thickness ≥ 3 mm, absence of hilum, heterogeneous enhancement, perfusion defects, and centripetal perfusion were significantly correlated with LN malignancy (*p* < 0.05). Benign LNs more often showed homogeneous enhancement and no perfusion defects. However, among metastatic LNs, 34.85%, 37.88%, 31.82%, and 50.00% presented with an L/S ≥ 2, the presence of hilum, homogeneous enhancement, and no perfusion defects, respectively. Additionally, 73.33% of benign LNs showed a cortical thickness ≥ 3 mm. Although centripetal enhancement generally indicates malignancy, mixed enhancement patterns were more prevalent in benign and malignant LNs. Furthermore, no significant differences were observed in vascular patterns between the two groups (*p* = 0.13).

**Figure 3:**
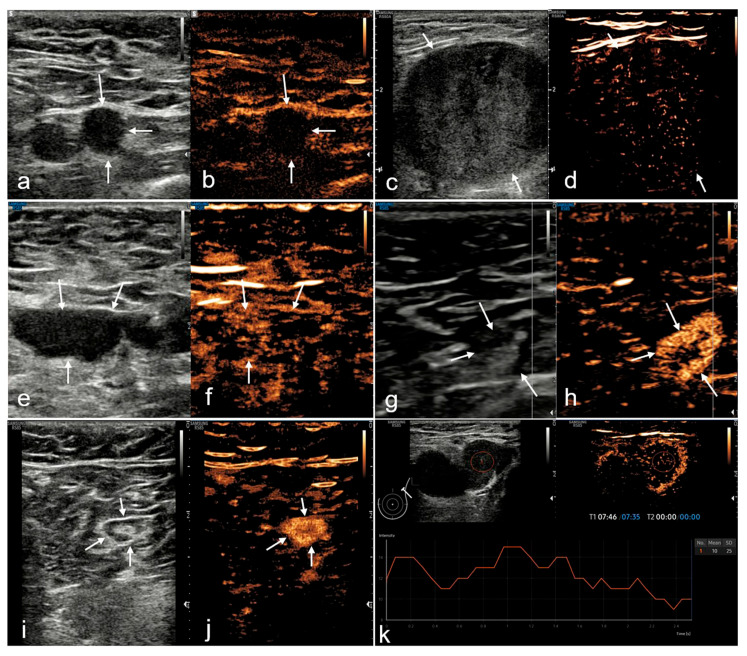
Enhancement patterns of LNs in the postvascular phase. The six sets of images depict gray-scale and contrast-enhanced images of axillary LNs in breast cancer patients. Note that none of the above LNs showed areas of persistent nonenhancement on CEUS. (**a**,**b**) The metastatic LN showed an essentially complete absence of enhancement in a 53-year-old woman (Pattern I) (arrows); (**c**,**d**) the metastatic LN showed starry hypo-enhancement in a 66-year-old woman (Pattern II) (arrows); (**e**,**f**) the metastatic LN showed heterogeneous hypo-enhancement with irregular unenhanced areas in a 63-year-old woman (Pattern III) (arrows); (**g**,**h**) the benign LN showed heterogeneous hyper-enhancement with unenhanced areas matched with the hilum in a 57-year-old woman (Pattern IV) (arrows); (**i**,**j**) the non-metastatic LN showed homogeneous hyper-enhancement in a 57-year-old woman (Pattern V) (arrows); (**k**) the metastatic LN showed annular enhancement in a 44-year-old woman (Pattern III). ROI was placed in the region of relative hypo-enhancement, and mean postvascular phase intensity (MPI) was calculated as 10 dB during 2.5 s by the time-intensity curve.

In the postvascular phase analysis, 80.00% (24/30) of benign LNs exhibited relative hyper-enhancement. Of the 66 metastatic LNs, 64 (96.97%) exhibited relative hypo-enhancement (*p* < 0.001), predicting metastasis with a sensitivity of 96.97%, specificity of 80.00%, positive predictive value (PPV) of 91.43%, negative predictive value of 92.31%, and accuracy of 91.67%. Relative hyper-enhancement suggested benign with a sensitivity of 80.00% and a specificity of 96.97%. Patterns I, II, and III were commonly observed in metastatic LNs (30.30%, 42.42%, and 24.24%), while Patterns IV and V were more prevalent in benign LNs (20.00% and 60.00%) (Figure 3). Notably, Pattern I demonstrated a specificity and PPV of 100% for diagnosing LNM, while Pattern IV displayed a specificity and PPV of 100% for benign nodules. Patterns III and IV (n = 24) were more frequently distributed in LNs with diameters ≥ 1 cm, accounting for 91.67% (22/24). In the subgroup with diameters < 1 cm (n = 14), Patterns I, II, and V, which showed a homogeneous distribution of the contrast agent, were more frequently observed, about 85.71% (12/14). Quantitative analysis showed that the MPI was significantly lower for malignant (median MPI, 12 dB) compared with benign (median MPI, 75 dB) LNs. The most effective threshold for MPI was a 32 dB signal intensity, which differentiated benign from metastatic LNs with a sensitivity of 92.42% and a specificity of 86.67%.

To evaluate the inter-observer agreement, the kappa values for enhancement homogeneity, perfusion defects, and perfusion pattern in the vascular phase, and enhancement degree and patterns in the postvascular phase were 0.71 (95% CI: 0.57, 0.85), 0.73 (95% CI: 0.59, 0.87), 0.72 (95% CI: 0.56, 0.88), 0.87 (95% CI: 0.76, 0.98), and 0.74 (95% CI: 0.64, 0.84), respectively.

### 3.3. Logistic Regression Analysis and ROC Curve Analysis

**Figure 4:**
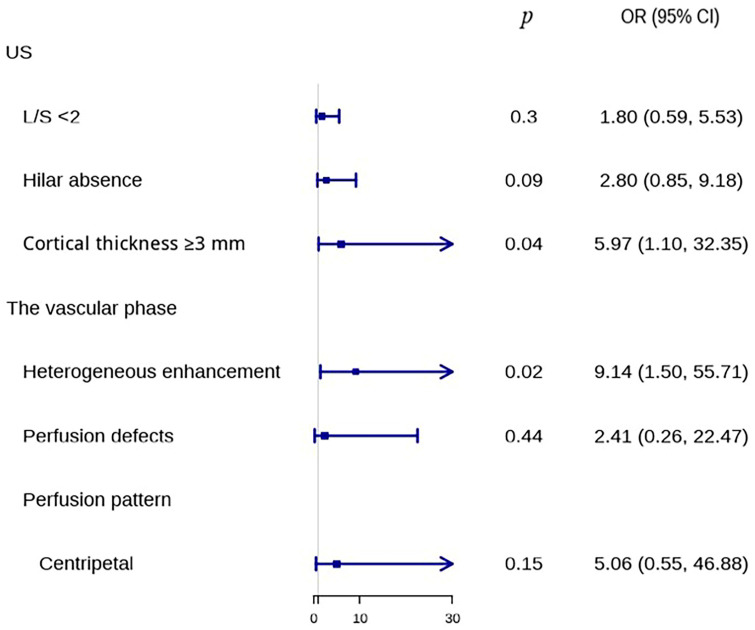
Forest plot of multivariate logistic regression of conventional US parameters and the vascular phase parameters to differentiate benign and metastatic LNs. CI, confidence interval; L/S, long-to-short diameter ratio; OR, odds ratio; US, ultrasound.

To evaluate the effectiveness of conventional US and the vascular phase in identifying benign and metastatic LNs, multivariate logistic regression analysis was used (Figure 4). Parameters significantly different between benign and metastatic LNs were included in the regression analysis, and diagnostic models were subsequently built. A cortical thickness ≥ 3 mm was identified as an independent factor for LNM using conventional US (odds ratio = 5.97, *p* = 0.04), whereas in the vascular phase group, heterogeneous enhancement was the independent factor (odds ratio = 9.14, *p* = 0.02).

**Figure 5:**
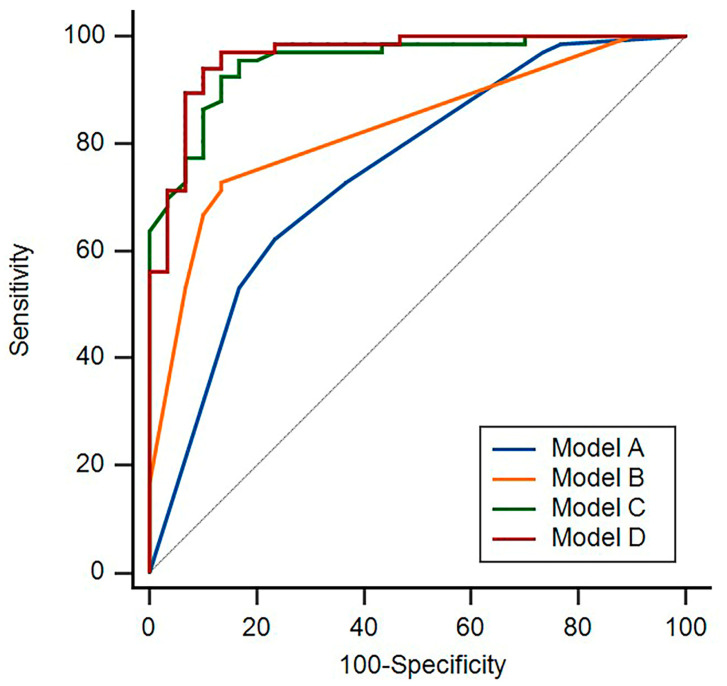
The receiver operating characteristic curves of four modalities for determining the benign or malignant status of LNs. Model A: comprising conventional US indicators, including L/S < 2, absence of the hilum, and cortical thickness ≥ 3 mm; Model B: comprising indicators in the vascular phase, including heterogeneous enhancement, perfusion defects, and centripetal perfusion; Model C: comprising quantitative indicator of the MPI in the postvascular phase; and Model D: combining seven indicators from Models A, B, and C, representing the combined predictive power of conventional US, CEUS vascular phase, and postvascular phase.

**Figure 6:**
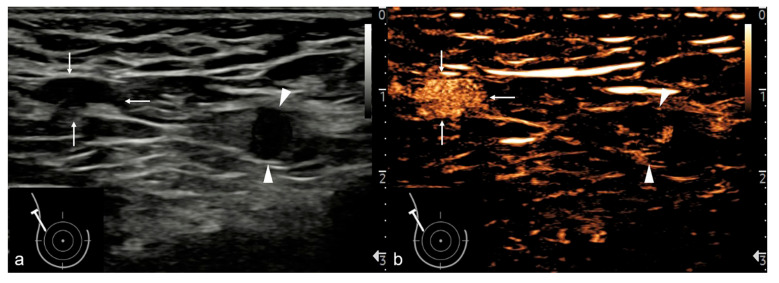
Representative case for the differentiation of benign and metastatic LNs. This was a 34-year-old woman with newly diagnosed breast cancer, presenting with multiple LNs in the right axilla: (**a**) B-mode US imaging of LN 1 (arrows) and 2 (arrowheads); (**b**) CEUS imaging of both LNs in the postvascular phase, showing homogeneous hyper-enhancement (Pattern V, MPI = 77 dB) of LN 1 (arrows) with a benign pathological diagnosis, and heterogeneous hypo-enhancement (Pattern III, MPI = 5 dB) of LN 2 (arrowheads), which proved to be metastatic. Note that neither LN showed areas of persistent nonenhancement on CEUS.

The predictive models for the conventional US, the vascular phase, and the postvascular phase of CEUS comprised the following parameters: Model A: comprising conventional US indicators, including L/S < 2, absence of the hilum, and cortical thickness ≥ 3 mm; Model B: comprising indicators in the vascular phase, including heterogeneous enhancement, perfusion defects, and centripetal perfusion; Model C: comprising a quantitative indicator of the MPI in the postvascular phase; and Model D: combining seven indicators from Models A, B, and C, representing the combined predictive power of conventional US, CEUS vascular phase, and postvascular phase. The diagnostic values for these models in differentiating benign and malignant LNs are reported in Table 4 and Figure 5. The parameter in the postvascular phase was more sensitive, specific, and accurate than conventional US and the vascular phase for LNM diagnosis, with an area under the curve (AUC) of 0.95 (95% CI: 0.89–0.99). The AUCs of Models C and D were significantly different from those of Models A and B (*p* < 0.001). Simultaneously, no significant difference was detected between the AUCs of Models A and B (*p* = 0.23) or Models C and D (*p* = 0.26). Utilizing the postvascular phase to identify benign and metastatic LNs is illustrated by a representative case in Figure 6.

## 4. Discussion

LNM detection is critical for cancer staging, treatment management decisions, and prognosis. Various imaging methods have been employed to assess LNM in BC patients. The application of CEUS for LNM diagnosis has rapidly evolved in recent years; however, the predictive value of CEUS parameters in the vascular phase, such as perfusion defects, heterogeneous enhancement, and centripetal or hybrid enhancement, remains limited [14,26,27]. This is consistent with our findings despite significant differences observed between benign and metastatic LNs in the univariate analysis. However, the postvascular phase of LNs has rarely been used to determine benign or malignant.

Korosh et al. [18] reported two types of contrast agents currently used for CEUS-based examination in practice: agents phagocytosed by the reticuloendothelial system, which demonstrates a “postvascular phase”, such as Sonazoid, and “blood pool” agents, such as Sonovue. Additionally, some researchers have demonstrated that Sonazoid microbubbles can be taken up by hepatic Kupffer cells, both in vivo and in vitro, demonstrating that the different postvascular imaging features of hepatic nodules result from the distribution of microbubbles in Kupffer cells [28,29,30]. Goldberg et al. [31] also observed microbubbles in LNs after contrast agent’s subcutaneous, submucosal, or parenchymal injections. Sonazoid has also been commonly used for detecting and characterizing sentinel LNs via subareolar injection [32,33,34,35]. However, no studies have observed the distribution of microbubbles in LNs after intravenous injection.

In our initial cases, persistent hyper-enhancement was observed in benign LNs after intravenous Sonazoid injection. In contrast, metastatic LNs commonly demonstrated relative hypo-enhancement, frequently with varying extents of unenhanced areas. This finding informed us of the presence of a postvascular phase in LNs when Sonazoid was injected intravenously. Our findings showed that 96.97% of metastatic LNs exhibited relative hypo-enhancement in the postvascular phase (*p* < 0.001), as confirmed by quantitative analysis with an AUC of 0.95. For nodules with heterogeneous hypo-enhancement, the area with the lowest enhancement during the postvascular phase typically offered a more suggestive indicator of possible tumor infiltration. Compared with the US and the vascular phase of CEUS, the postvascular phase was more sensitive, specific, and accurate in diagnosing LNM, which could help to adjust the N staging in clinical TNM classification, especially in the assessment of clinically nonpalpable nodules, thereby influencing subsequent surgical or adjuvant therapeutic strategies. Additionally, in the subgroup study of LN diameter, we found that smaller LNs more often showed uniform no/low/high enhancement, and as the diameter increased, focal perfusion abnormalities such as annular enhancement and other inhomogeneous enhancements were more likely to be observed. Notably, postoperative histopathological examination of LNs exhibiting Pattern IV often demonstrated a benign nature; thus, the ROI was placed in areas with hyper-enhancement to yield a more suggestive MPI for these specific LNs.

It is necessary to perform CEUS evaluations in larger patient cohorts in the future, such as multicenter studies or external validation. In addition, the impact of LN size on the diagnostic efficacy of indicators in the CEUS postvascular phase needs to be investigated.

This study had several limitations. First, US-guided histological biopsy, rather than surgical pathological examination, was performed in most LNs, potentially leading to false-negative outcomes. Of the LNs with benign puncture results (n = 30), 12 were included directly in the follow-up cohort without obtaining surgical pathology. Thus, the possibility of microinfiltrates or benign changes resulting from adjuvant therapy cannot be eliminated. Second, the enhancement degree and patterns of CEUS were subjectively determined, making it difficult to identify the ultrasonic features of some small LNs. Third, our study was subject to selection bias, as it selectively enrolled patients with undetermined LNs detected by conventional US. Fourth, the formation of the postvascular phase after intravenous Sonazoid injection and its distribution in the LNs require further investigation.

## 5. Conclusions

In conclusion, our study showed that the degree of enhancement and the MPI in the postvascular phase can effectively differentiate metastatic and benign LNs in patients with BC with excellent diagnostic reliability. The postvascular phase could be an essential phase for CEUS and provide a potential solution to the challenging clinical problem of assessing nodal status.

## Figures and Tables

**Table 1:** Comparison of the differences between the two phases of contrast-enhanced ultrasound.

Comparison	Injection Method	Contrast Agent	Observe Time (After Injection)	Observed Indicator
The vascular phase	Intravenous	Sonazoid	0–1 min	Enhancement homogeneity; perfusion defects; perfusion pattern
The postvascular phase	Intravenous	Sonazoid	7–9 min	Enhancement degree; enhancement pattern; mean postvascular phase intensity

**Table 2:** Demographic and clinical characteristics of the study sample.

Variables	Total
No. of patients	90
No. of lymph nodes	96
Age, years	54.44 ± 12.29
Lymph nodes	
Diameter of major axis, cm	1.84 ± 0.85
Nodal status	
Non-metastasis	30 (31.25%)
Metastasis	66 (68.75%)
Location	
Axilla	80 (83.33%)
Neck	13 (13.54%)
Pectoral muscles	3 (3.13%)
Breast operation	
Pre-operation	64 (71.11%)
Post-operation	26 (28.89%)
Tumor type	
Carcinoma in situ	3 (3.33%)
Invasive (No special type)	83 (92.22%)
Other	4 (4.44%)

Data are presented as mean ± standard deviation.

**Table 3:** Sonographic features of benign and metastatic lymph nodes.

	Metastasis *(n* = 66)	Non-Metastasis (*n* = 30)	χ^2^	*p*
Conventional US				
L/S			8.44	0.004 **
<2	43 (65.15%)	10 (33.33%)		
≥2	23 (34.85%)	20(66.67%)		
Hilum			12.41	<0.001 ***
Absent	41 (62.12%)	7 (23.33%)		
Present	25 (37.88%)	23 (76.67%)		
Cortical thickness, mm			9.95	0.002 **
≥3	64 (96.97%)	22 (73.33%)		
<3	2 (3.03%)	8 (26.67%)		
Vascular pattern			4.04	0.13
Peripheral or mixed	48 (72.73%)	18 (60.00%)		
Hilar	7 (10.60%)	8 (26.67%)		
None	11 (16.67%)	4 (13.33%)		
The vascular phase				
Enhancement homogeneity			22.68	<0.001 ***
Heterogeneous	45 (68.18%)	4 (13.33%)		
Homogeneous	21 (31.82%)	26 (86.67%)		
Perfusion defects			14.90	<0.001 ***
Present	33 (50.00%)	2 (6.67%)		
Absent	33 (50.00%)	28 (93.33%)		
Perfusion pattern			7.93 ^b^	0.02 *
Centripetal	14 (21.21%)	1(3.33%)		
Hybrid	51 (77.27%)	26 (86.67%)		
Centrifugal	1 (1.52%)	3 (10.00%)		
The postvascular phase				
Degree of enhancement			58.04	<0.001 ***
Relative hypo-enhancement	64 (96.97%)	6 (20.00%)		
Relative hyper-enhancement	2 (3.03%)	24 (80.00%)		
Patterns of enhancement				
Pattern I	20 (30.30%)	0		
Pattern II	28 (42.42%)	4 (13.33%)		
Pattern III	16 (24.24%)	2 (6.67%)		
Pattern IV	0	6 (20.00%)		
Pattern V	2 (3.03%)	18 (60.00%)		
MPI, dB ^a^	12 (5, 20)	75 (47, 93)	97.50 ^c^	<0.001 ***

^a^ Data are expressed as median (interquartile range); ^b^ Fisher’s exact test; ^c^ Mann–Whitney U test; * *p* < 0.05; ** *p* < 0.01; *** *p* < 0.001. L/S, long-to-short diameter ratio; MPI, mean postvascular phase intensity; US, ultrasound.

**Table 4:** The diagnostic value of different modalities in differentiating benign and malignant LNs.

Model	AUC (95% CI)	Sensitivity (%)	Specificity (%)	PPV (%)	NPV (%)	Accuracy (%)
A	0.75 (0.65–0.83)	62.12	76.67	85.42	47.92	66.67
B	0.83 (0.74–0.90)	72.73	86.67	92.31	59.09	77.08
C	0.95 (0.89–0.99)	92.42	86.67	93.85	83.87	90.63
D	0.96 (0.90–0.99)	93.94	90.00	95.38	87.10	92.71

Model A: Comprising conventional US indicators, including L/S < 2, absence of the hilum, and cortical thickness ≥3 mm; Model B: comprising indicators in the vascular phase, including heterogeneous enhancement, perfusion defects, and centripetal perfusion; Model C: comprising quantitative indicator of the mean postvascular phase intensity; and Model D: combining seven indicators from Models A, B, and C, representing the combined predictive power of conventional US, CEUS vascular phase, and postvascular phase. AUC, area under the curve; CI, confidence interval; NPV, negative predictive value; PPV, positive predictive value.

## Data Availability

The data presented in this study are available upon request from the corresponding author. The data are not publicly available due to ethical restrictions.
